# Effect of cerebral oxygen saturation monitoring in patients undergoing superficial temporal anterior-middle cerebral artery anastomosis for ischemic Moyamoya disease: a prospective cohort study

**DOI:** 10.3389/fneur.2023.1226455

**Published:** 2023-09-21

**Authors:** Xuanling Chen, Xuewei Qin, Jing Wang, Rong Wang, Xiangyang Guo, Lan Yao

**Affiliations:** ^1^Department of Anesthesiology, Peking University International Hospital, Beijing, China; ^2^Department of Neurosurgery, Peking University International Hospital, Beijing, China; ^3^Department of Neurosurgery, Tiantan Hospital, Capital Medical University, Beijing, China; ^4^Department of Anesthesiology, Peking University Third Hosptial, Beijing, China

**Keywords:** ischemic MMD, superficial temporal artery-middle cerebral artery anastomosis, regional cerebral oxygen saturation (rSO_2_), anesthesia management, mean arterial pressure

## Abstract

**Objective:**

Regional cerebral oxygen saturation (rSO_2_) is linked with blood pressure. This study evaluated the influence of perioperative rSO_2_ monitoring on the prognosis of ischemic Moyamoya disease (MMD) patients undergoing anastomosis surgery.

**Methods:**

In this prospective cohort, patients with unilateral ischemic MMD of Suzuki stage ≥3 were included. The decision of rSO_2_ was made by the clinician and the patient. The rSO_2_ group maintained intraoperative rSO_2_ levels through the modulation of blood pressure, inhaled oxygen concentration, carbon dioxide in arterial blood, and red blood cell transfusion. The non-rSO_2_ group used conventional anesthesia practices. Perioperative mean arterial pressure (MAP), rSO_2_ values, neurological complications, and postoperative results were assessed.

**Results:**

A total of 75 eligible patients were categorized into a rSO_2_ monitoring group (*n* = 30) and a non-rSO_2_ monitoring group (*n* = 45). For the rSO_2_ group, the preoperative rSO_2_ was significantly lower on the affected side (*P* < 0.05). After anastomosis, this value notably increased (*P* = 0.01). A moderate relationship was observed between perioperative rSO_2_ and MAP before, during, and after surgery, with correlation coefficients (*r*) of 0.536, 0.502, and 0.592 (*P* < 0.05). Post-surgery MAP levels differed between the groups, with the rSO_2_ group showing decreased levels compared to pre-surgery and the non-rOS_2_ group displaying elevated levels. Notably, the rSO_2_ group reported shorter hospitalizations and decreased neurological complications. Patients with a hypertension history found postoperative MAP influencing hospital stay duration.

**Conclusion:**

Perioperative rSO_2_ surveillance enhanced cerebral perfusion and minimized postoperative complications in ischemic MMD patients. Thus, rSO_2_ monitoring is advocated for MMD patients undergoing vascular anastomosis.

## 1. Introduction

Moyamoya disease (MMD) is a chronic vascular brain disorder marked by progressive stenosis or occlusion of crucial cerebral arteries. Its clinical presentation is varied, including epilepsy, cognitive dysfunction, headache, and cerebral ischemia (1). The disease affects 1.14 out of 100,000 people in China (2), with women being twice as susceptible as men (3). It typically presents in children at ~5 years of age and adults in their 40s (4).

Surgery is the predominant treatment for MMD (5), with the superficial temporal anterior-middle cerebral arterial anastomosis being the standard approach for ischemic MMD (6). This surgery promotes blood supply from the unaffected parietal branch of the superficial temporal artery to the ischemic brain hemisphere (7), enhancing perfusion and setting up collateral circulation (8). However, perioperative complications such as cerebral hemorrhage and infarction remain a concern. It is essential to balance oxygen supply and demand in the brain's affected area during the perioperative period and swiftly address intraoperative cerebral hypoxia to prevent stroke (9). Traditionally, anesthesiologists have relied on experience for perioperative management, using preoperative mean arterial pressure (MAP), partial pressure of carbon dioxide in arterial blood (PaCO_2_), and other cerebral perfusion indicators. The perioperative MAP should be kept roughly 10% above the preoperative level to boost cerebral perfusion during vascular anastomosis (10). However, post-anastomosis, the affected brain region's blood flow can surge, risking vessel rupture and hemorrhage (11). Notably, if the empirical systolic blood pressure remains under 130 mmHg post-surgery and postoperative brain tissue perfusion is not improved (12), it fails to ameliorate the affected side's cerebral tissue perfusion. This can lead to complications such as hyperperfusion syndrome (CHS) (13), cerebral hemorrhage (14), and infarction (15). Thus, managing blood pressure is paramount during the perioperative phase (16).

Regional cerebral oxygen saturation (rSO_2_) offers non-invasive, real-time monitoring of local cerebral blood perfusion. It has been employed in various surgeries, including cardiac, orthopedic, and neurosurgery, displaying remarkable accuracy in gauging oxygen saturation and minimizing postoperative neurological complications (17). Prior research indicates that rSO_2_ reliably represents cerebral perfusion in MMD patients and can predict postoperative outcomes such as delirium, cerebrovascular reactive delirium (18), and CHS (19, 20). However, the utilization of rSO_2_ for perioperative blood pressure monitoring in MMD patients remains under-explored (1).

This study aimed to assess the impact of rSO_2_ monitoring in MMD patients undergoing unilateral superficial temporal artery to middle cerebral artery branch anastomosis.

## 2. Methods

### 2.1. Study design and surgical procedure

#### 2.1.1 Study design and participants

This prospective cohort study included 110 patients with ischemic MMD who underwent direct anastomosis between the superficial temporal artery and the middle cerebral arterial branch for the first time between January 2019 and July 2021 in the Neurosurgery Department of Peking University International Hospital. The ethics committee of the university approved the study, and all patients provided signed informed consent for their participation.

The inclusion criteria were as follows: (1) patients with unilateral ischemic MMD seen on cerebral angiography; (2) patients without intracranial space-occupying lesions, cerebral hemorrhage, aneurysms, massive cerebral infarction, and other related diseases detected on CT imaging; (3) patients aged between 18 and 65 years and with any gender; (4) patients with ASA physical status classification scores of I and II; (5) patients with the Suzuki staging of ≥3 calculated on digital subtraction angiography (DSA) and the score of Mini-mental State Examination (MMSE) of >24 points; and (6) patients who furnished signed informed consent. The exclusion criteria were as follows: (1) patients who experienced severe immune system diseases or severe organ dysfunction, such as heart, liver, and kidney dysfunctions; (2) patients who underwent prior craniocerebral surgery; (3) patients with hemorrhagic MMD; (4) patients who had severe stenosis or occlusion of the subclavian artery and carotid artery; (5) patients with frontal skin infections in which the measuring of rSO_2_ could not have been done; (6) patients with bilateral ischemic MMD; and (7) changed surgical protocol or patients who withdrew voluntarily.

Seventy-five patients were included in the study analysis. The decision was made by the clinician and the participants whether monitoring of rSO_2_ is needed or not. The participants were divided into two groups according to whether rSO_2_ was monitored or not: the rSO_2_ monitoring group (the rSO_2_ group) and the non-rSO_2_ monitoring group (the non-rSO_2_ group).

### 2.2. Anesthesia and monitoring methods

#### 2.2.1. Anesthesia protocol

Midazolam at a dosage of 0.02 mg/kg, propofol at a dosage of 1.5 mg/kg, sufentanil at a dosage of 0.4 μg/kg, and rocuronium at a dosage of 1 mg/kg were used for inducing anesthesia in both groups. When the bispectral index (BIS) reached 40–60 (the electrode was placed on the forehead contralateral side), endotracheal intubation and mechanical ventilation were connected. The end-tidal expiratory carbon dioxide pressure (PetCO_2_) was maintained between 35 and 45 mmHg by adjusting tidal volume and respiratory rate, and the inhaled oxygen concentration was adjusted to 60%. Anesthesia was maintained by intravenous infusion of propofol at the rate of 3–4 mg/(kg·h), remifentanil at the rate of 0.1–0.2 μg/(kg·min), continuous inhalation of sevoflurane at a percentage concentration of 0.8%−1.0%, and BIS was maintained between 40 and 60. During the operative procedure, rocuronium was given intravenously, and 10 μg sufentanil was given at the time of suturing the scalp. After completion of the scalp suture, all anesthetic drugs were withdrawn.

#### 2.2.2. Monitoring of blood pressure and medications for blood pressure adjustment

Fluid access was established, and the Allen test was negative on the right/left hand. Subsequently, the radial artery puncture catheterization was performed, and the MAP (intraoperative MAP), ECG, SPO_2_, body temperature, and BIS were determined. The rSO_2_ (Medtronic 5100C, America) was monitored in the rSO_2_ group. In the rSO_2_ group, rSO_2_ and MAP were monitored 1 day before surgery in the ward. The same trained anesthesiologist performed all rSO_2_ monitoring and postoperative follow-up. Method of rSO_2_ monitoring: alcohol was applied to the forehead for sterilization and dehydration. Then, patches for measuring rSO_2_ were placed on the forehead 1 cm away from the middle of the forehead and 1–2 cm above the eyebrow arch, and the rSO_2_ monitor was connected. The baseline rSO_2_ values were calibrated and monitored continuously for 3 h after stabilization of values for 5 min. MAP was measured from the right upper limb, with an interval of 5 min, and monitored for 3 h. The monitoring was performed on alternate days after entering the operating room. During surgery, blood pressure was adjusted in the rSO_2_ group with the observation of rSO_2_. The objective of monitoring rSO_2_ was to maintain no more than a 20% decrease in the baseline value or no more than a 55% decrease in the baseline absolute value during surgery.

When the rSO_2_ was lower than this level, the following treatment measures were adopted (21): (1) The connection and position of the rSO_2_ patch were checked; (2) The blood pressure was increased; (3) The concentration of inhaled O_2_ was raised; (4) If the above treatment measures did not improve the rSO_2_, blood gas was analyzed, and if anemia was present (<80 g/L), 2–4 U suspended red blood cells were transfused based on the oxygen saturation. The rSO_2_ was monitored for three consecutive days following the surgery. On D_1_, D_2_, and D_3_, all data were collected by the same anesthesiologist. In the non-rSO_2_ group, the anesthesiologist maintained the MAP before vascular anastomosis ~10% above the baseline value of the preoperative MAP. The intraoperative MAP was collected by the monitor. If the blood pressure decreased during the operation, ephedrine of 3–6 mg intravenously was administered to achieve the blood pressure rapidly, and also, noradrenaline was continuously administered to maintain the target blood pressure level. The endotracheal cannula was removed after the patient recovered from anesthesia, and the patient returned to the ward. For three consecutive days after surgery (D_1_, D_2_, and D_3_), patients in both groups were continuously monitored for 8 h (8 am to 4 pm) based on MAP or rSO_2_.

### 2.3. Indicators that determine the outcome

#### 2.3.1. Outcome indicators

The perioperative period (22) is referred to 7 days before surgery to 7 days after surgery. The outcome indicators were the postoperative cerebral hemorrhage, cerebral infarction, and incidence of CHS in the two groups.

The clinical manifestations of cerebral hemorrhage and CHS were similar, such as postoperative headache, seizure, and focal neurological deficit. (1) Diagnosis of cerebral hemorrhage: new intracranial blood was seen on a postoperative craniocerebral CT scan. (2) Diagnosis of CHS: (a) The diagnosis was done by observation of local neurological dysfunction and epilepsy. (b) CT/MRI perfusion imaging and SPECT scans showed that blood perfusion at the bypass site increased from the lowest value before surgery to the highest value after surgery and was locally clustered. (c) The diagnosis was performed by excluding local muscle swelling and compression (23). (3) Diagnosis of cerebral infarction: postoperative craniocerebral CT or MRI images showed new cerebral ischemic lesions. All patients received an MRI for outcome evaluation. The postoperative complications of all patients were analyzed by the same senior neurosurgeon and radiologist.

### 2.4. Data source and evaluation

Data were obtained from the medical records, anesthesia information system, and rSO_2_ monitors of Peking University International Hospital. The general characteristics of 75 patients were collected, and operation time and MAP values were recorded for the preoperative period (3 h on the day before the surgery), intraoperative period (from the time when the skin was incised to the time of the end of suturing), postoperative period (from the end of the surgery to the time when leaving the surgery room), and postoperative three consecutive days (D_1_, D_2_, and D_3_). Hemoglobin content from the blood gas analysis before and after surgery was recorded for the two groups. The perioperative rSO_2_ mean values (D_1_, D_2_, and D_3_) were recorded in the rSO_2_ group. The data of cerebral infarction, CHS, cerebral hemorrhage, reoperation, and death were recorded from the postoperative period to the time of discharge.

### 2.5. Statistical analyses

SPSS version 20.0 statistical software was used to analyze the data. The Shapiro–Wilk test evaluated the normal distribution. The measurement data were expressed as mean ± standard deviation (mean ± SD). If they did not fit the normal distribution, the quartile method M (P25 and P75) was used. The intra-group comparison was performed by *t*-test and repeated measure analysis of variance between the two groups. For comparing the measurement data between groups, repeated measurement analysis of variance was used for complex conditions. The least significant difference (LSD) method was used for the comparison between the groups and the Kruskal–Wallis H method was used for testing if the application conditions were not met. Counting data were expressed as frequencies and percentages and compared using the chi-square tests. The Kolmogorov–Smirnov test was used to evaluate the data distribution. The Pearson method was used for correlation analysis. An alpha value of 0.05 and a *P*-value of <0.05 were considered to be statistically significant differences.

## 3. Results

### 3.1. The flow chart for the selection of patients

**Figure 1:**
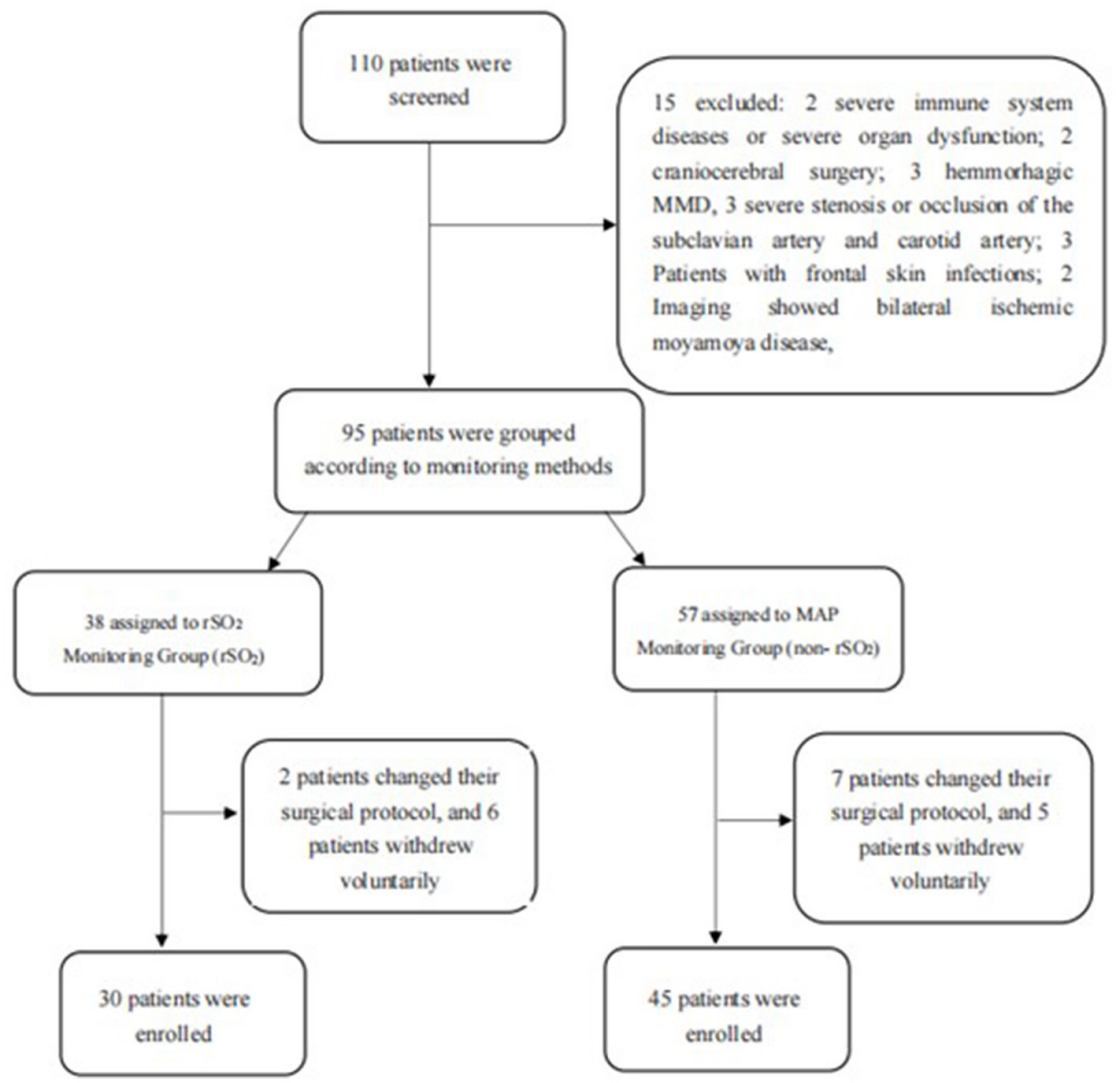
Flow chart for the patient selection.

In this study, 110 patients were enrolled, and 15 patients were excluded due to severe immune system diseases or severe organ dysfunction (*n* = 2), craniocerebral surgery (*n* = 2), hemorrhagic MMD (*n* = 3), severe stenosis or occlusion of the subclavian artery and carotid artery (*n* = 3), frontal skin infections (*n* = 3), and bilateral ischemic Moyamoya disease. Finally, 95 patients were screened and divided into the rSO_2_ group (*n* = 38) and the non-rSO_2_ group (*n* = 57) according to whether the rSO_2_ was monitored or not. In the rSO_2_ group, the surgical protocol was changed in two patients, and six patients withdrew voluntarily from the study. In the non-rSO_2_ group, the surgical protocol was modified in seven patients, and five patients withdrew voluntarily from the study. Finally, 38 patients were included in the rSO_2_ group and 45 in the non-rSO_2_ group. The flow chart for the selection of patients is shown in Figure 1.

### 3.2. General characteristics of patients

Seventy-five patients were included in the analysis, and all underwent direct anastomosis of the unilateral superficial temporal artery and middle cerebral arterial branch. The mean age of the patients in the rSO_2_ group was 47.33 ± 7.52 years, and the preoperative MAP was 97.28 ± 11.67 mmHg. The mean age of the patients in the non-rSO_2_ group was 46.30 ± 7.80 years, and the preoperative MAP was 98.57 ± 9.92 mmHg. A statistical difference in the general characteristics of patients was found between the two groups, except for gender (*P* > 0.05), as shown in Table 1.

**Table 1:** General characteristics of patients in both groups.

**Parameters**	**rSO_2_ group (*n* = 30)**	**Non-rSO_2_ group (*n* = 45)**	**P-value**
Age (mean ± SD), years	46.30 ± 7.80	47.33 ± 7.52	0.567
**Sex**			
Male, *n* (%)	19 (63.33)	11 (24.44)	0.000
Female, *n* (%)	11 (36.67)	34 (75.56)	
BMI (mean ± SD), kg/m^2^	26.22 ± 3.01	26.09 ± 2.20	0.821
Operation time (mean ± SD), min	275.97 ± 22.30	272.76 ± 33.97	0.650
**Operative side**			
Left	13 (43.33)	18 (40)	0.774
Right	17 (56.67)	27 (60)	
MAP (mean ± SD), mm Hg	98.57 ± 9.92	97.28 ± 11.67	0.620
**Arterial systolic blood pressure**, ***n*** **(%)**			
100–140 mmHg (1)	16 (53.33)	21 (46.67)	0.572
141–180 mmHg (2)	14 (46.67)	24 (53.33)	
Heart rate (mean ± SD), bpm	77.90 ± 8.66	78.04 ± 9.69	0.948
SpO_2_ (mean ± SD), %	98.93 ± 1.59	98.78 ± 1.53	0.673
PaO_2_ (mean ± SD), mm Hg	86.33 ± 5.14	84.60 ± 4.32	0.119
PaCO_2_ (mean ± SD), mm Hg	36.79 ± 2.59	36.50 ± 3.49	0.701
Hb (before surgery; mean ± SD), g/dl	117.27 ± 14.04	117.00 ± 10.83	0.926
Hb (after surgery; mean ± SD), g/dl	114.63 ± 13.75	112.33 ± 18.73	0.566

### 3.3. Multiple factors influencing the length of stay (multi-factor analysis)

The length of stay in the rSO_2_ group was 7.73 ± 2.23 days and in the non-rSO_2_ group was 9.04 ± 2.68 days with a statistically significant difference (*P* = 0.03). The length of stay in the two groups was correlated to rSO_2_ monitoring, history of hypertension, preoperative PaCO_2_, preoperative Hb, time of surgery, postoperative MAP D_1_, MAP D_2_, MAP D_3_, and preoperative PaO_2_. Utilizing the rSO_2_ monitored could have shortened the length of stay (*P* = 0.042). Patients with a history of hypertension, postoperative MAP D_1_, MAP D_2_, and MAP D_3_ affected the length of stay, and the rest were not significantly correlated, as shown in Table 2.

**Table 2:** Multivariate analysis for the length of stay.

**Parameters**	***B*-value**	***t-*value**	**P-value**	**95.0% confidence interval of** ***B***	
				**Lower limit**	**Upper limit**
Use of rSO_2_ or not	−1.233	−2.068	0.042	−2.423	−0.044
History of hypertension	1.286	2.186	0.032	0.112	2.460
Preoperative PaCO_2_	0.03	0.39	0.696	0.89	1.2
Preoperative Hb	0.002	0.094	0.925	0.96	1.04
Time of surgery	0.004	0.46	0.645	0	0.31
**Postoperative**					
MAP D_1_	−0.057	−2.108	0.035	0.9	1
MAP D_2_	−0.064	−2.375	0.018	0.89	0.99
MAP D_3_	−0.099	−2.774	0.006	0.84	0.97
Preoperative PaO_2_	0.081	1.539	0.124	0.98	1.2

### 3.4. Perioperative mean arterial pressure

No significant difference in preoperative MAP was found between the two groups perioperatively (*P* = 0.62). Within the rSO_2_ group, the intraoperative MAP and postoperative MAP decreased significantly compared to the preoperative MAP (*P* < 0.01). Within the non-rSO_2_ group, the intraoperative and postoperative MAPs differed significantly from the preoperative MAP (*P* = 0.00), as shown in Table 3.

**Table 3:** Comparison of MAP of perioperative periods between the rSO_2_ group and the non-rSO_2_ group (mean ± SD).

**Parameters**	**rSO_2_ (*n* = 30)**	**non-rSO_2_ (*n* = 45)**	***t*-value**	**P-value**
Preoperative MAP	98.57 ± 9.92	97.28 ± 11.67	−0.498	0.620
Intraoperative MAP	91.00 ± 9.22	102.26 ± 7.21	5.922	0.000
Postoperative MAP	93.53 ± 6.90	104.05 ± 8.22	5.779	0.000
MAP D_1_	94.76 ± 8.99	99.45 ± 10.70	1.980	0.002
MAP D_2_	93.99 ± 9.59	100.12 ± 10.45	2.564	0.012
MAP D_3_	93.81 ± 7.76	99.75 ± 8.11	3.159	0.002

### 3.5. Perioperative rSO_2_

The preoperative rSO_2_ in the rSO_2_ group was significantly lower on the affected side than on the healthy side (*P* = 0.00). The rSO_2_ of the affected side was higher than that of the healthy side after vascular anastomosis, and the difference was statistically significant (*P* = 0.01). The rSO_2_ on the affected side was higher than that on the same side before surgery (*P* < 0.05).

The rSO_2_ of D_1_, D_2_, and D_3_ on the affected side were significantly higher than those on the healthy side for 3 days after surgery (*P* < 0.01; Table 4).

**Table 4:** Comparison of the rSO_2_ between the affected side and the healthy side in the rSO_2_ group at each time of the perioperative period (mean ± SD).

**Parameters**	**Healthy side (*n* = 30)**	**Affected side (*n* = 30)**	***t*-value**	**P-value**
Preoperative rSO_2_	66.87 ± 7.82	61.77 ± 7.84^*^	5.391	0.000
Intraoperative rSO_2_	69.13 ± 6.34	71.48 ± 6.10^*^	−2.670	0.012
Postoperative rSO_2_	71.07 ± 7.12	71.87 ± 8.47	−0.582	0.565
rSO_2_ D_1_	68.87 ± 5.16	73.03 ± 5.64	3.358	0.002
rSO_2_ D_2_	68.67 ± 5.45	75.21 ± 4.50	5.052	0.000
rSO_2_ D_3_	69.20 ± 5.94	75.09 ± 3.84	4.771	0.000

^*^Statistical significance difference.

### 3.6. Relationship between rSO_2_ and MAP during the perioperative period

**Figure 2:**
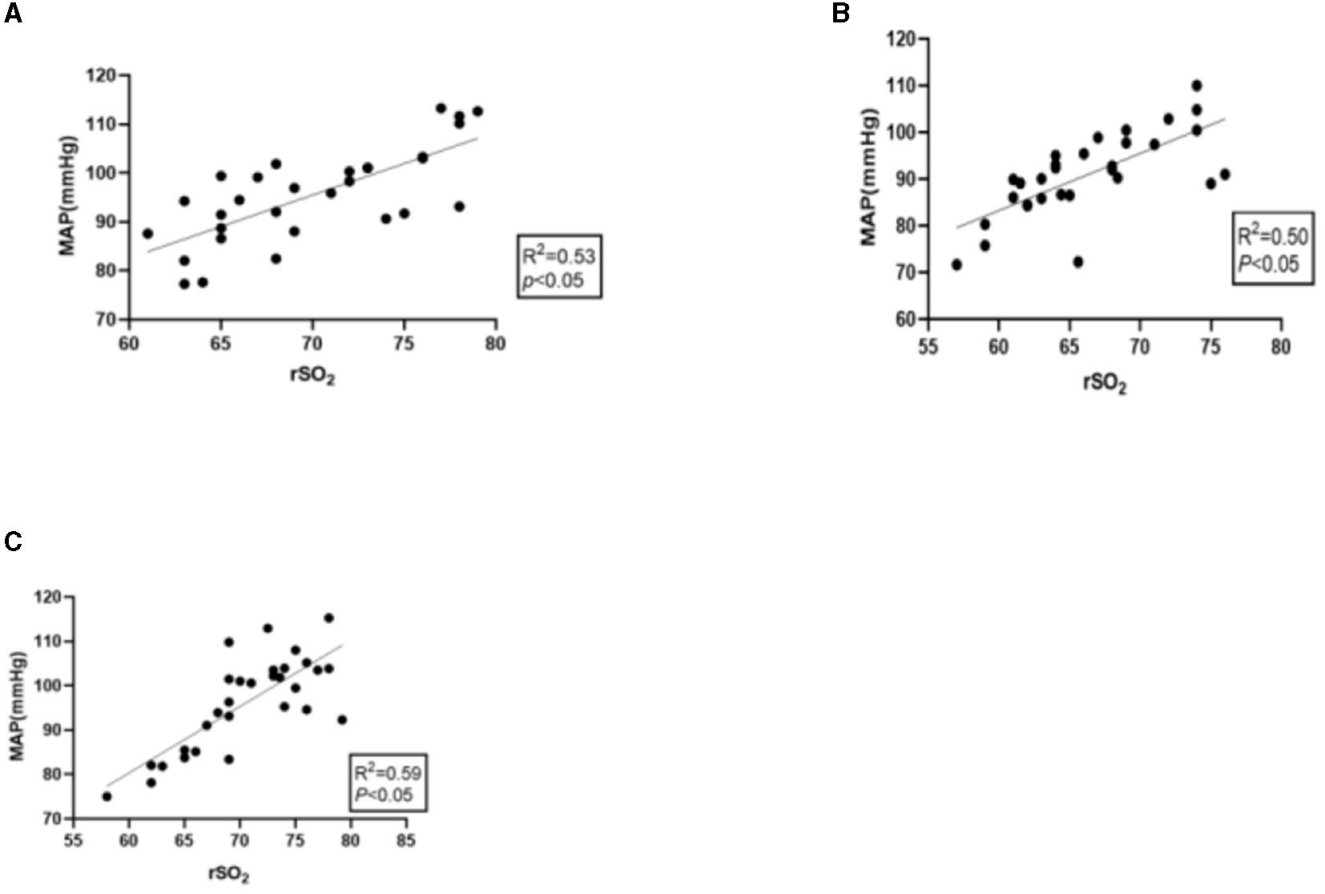
**(A)** Correlation between preoperative MAP and mean rSO_2_. **(B)** Correlation between intraoperative MAP and mean rSO_2_. **(C)** Correlation between postoperative MAP and mean rSO_2_.

The mean value of rSO_2_ showed a moderately positive correlation to the corresponding MAP before, during, and after surgery, and the correlation coefficients (r) were 0.536, 0.502, and 0.592, respectively (*P* < 0.05), as shown in Figures 2A–C.

### 3.7. Neurological complications

One case had CHS (3.3%), and one case experienced cerebral hemorrhage (3.3%) in the rSO_2_ group between the time after surgery and the time of discharge. Two cases had cerebral hemorrhage (4.4%), and 10 cases had CHS (33.3%) in the non-rSO_2_ group. No cerebral infarction, reoperation, or death occurred in the two groups. Fisher's test showed that neurological complication in the rSO_2_ group were significantly less than those in the non-rSO_2_ group (*P* = 0.036).

### 3.8. Logistic regression analysis of complications, use of rSO_2_, and blood pressure at different time periods.

Postoperative complications such as CHS and cerebral hemorrhage in patients with ischemic MMD were correlated with rSO_2_ and the management of MAP during and for three consecutive days after surgery (D_1_, D_2_, and D_3_; *P* < 0.05), as shown in Table 5.

**Table 5:** Multivariate logistic regression analysis of MMD postoperative complications with rSO_2_ and MAP at different time periods.

**Parameters**	***B***	***T***	**Sig**	***B*** **95% (CI)**	
				**Upper Limit**	**Lower Limit**
rSO_2_ monitoring	0.075	1.97	0.049	1	1.16
Preoperative MAP	0.09	2.495	0.013	1.02	1.17
Intraoperative MAP	0.113	2.723	0.006	1.03	1.21
Postoperative MAP	0.117	3.016	0.003	1.04	1.21
MAP D_1_	0.259	3.605	<0.001	1.13	1.49
MAP D_2_	0.193	3.701	<0.001	1.09	1.34
MAP D_3_	0.200	3.493	<0.001	1.09	1.37

## 4. Discussion

Blood pressure is regulated to ensure good cerebral perfusion when the brain region is affected. However, blood pressure cannot directly reflect the blood perfusion in encephalopathy, which may be an indirect indicator (24). The rSO_2_ is the measure of blood oxygen saturation in local tissues, including the brain tissue, and is monitored non-invasively.

Many previous studies (4, 25–27) found that frequent intraoperative monitoring of rSO_2_ had a certain predictive value of intraoperative cerebral ischemia and postoperative neurological complications. Samra et al. (28) found that when rSO_2_ was reduced by <20% of the baseline value, the occurrence of postoperative neurological complications was significantly reduced, and the rSO_2_ level had high sensitivity (80%) and specificity (82.2%) in predicting neurological complications.

The patients in the rSO_2_ group were monitored for rSO_2_ on the day before surgery, and the rSO_2_ value on the affected side was lower than that on the healthy side, suggesting that vascular lesions could have led to abnormal cerebral perfusion. The sensitivity of rSO_2_ level in monitoring cerebral perfusion had another viewpoint. The results of the present study showed a moderate positive correlation between perioperative rSO_2_ and MAP, which suggests that empirical blood pressure management is necessary. In a normal population with conditions of reasonable blood volume, no serious cardiac dysfunction, and no serious anemia, the improvement of cerebral perfusion can be achieved by adjusting the patient's blood pressure using only vasoactive drugs. However, in patients with ischemic MMD, it might not be beneficial to simply elevate blood pressure due to the fragile vascular mass. At this point, an individualized blood pressure regulation program may be more appropriate for such patients when cerebral perfusion decreases and rSO_2_ decreases. In addition to increasing blood pressure, cerebral perfusion in the affected area of the brain can also be improved through comprehensive treatments, such as increasing inhaled O_2_ concentration, adjusting PaCO_2_, and transfusing red blood cells (29) to reduce the incidence of CHS and cerebral hemorrhage caused by the elevated blood pressure.

After vascular anastomoses, rSO_2_ on the affected side increased even more than that on the healthy side, demonstrating that blood from the superficial temporal artery on the affected side entered the cerebral ischemic area and improved the cerebral tissue blood perfusion. The rSO_2_ was monitored continuously for 3 days after surgery in the rSO_2_ group, and the value of rSO_2_ on the diseased side was similar to the value at the time of vascular opening after vascular anastomosis, which may have been due to the poor compensatory capacity of cerebral vessels in the affected area, the increased cerebral blood flow shunt, and the ischemic area reaching a new equilibrium point after a certain period. The rSO_2_ value could reflect the cerebral perfusion on the affected side in real-time and let the anesthesiologist adjust accordingly, which could reduce the occurrence of postoperative neurological complications, ensuring surgical benefit, and improving the postoperative outcome of patients (all patients in the rSO_2_ group received an intraoperative reduction of BP due to the rSO_2_ measurement). The results of He et al. (29) show that the length of hospital stay in the non-rSO_2_ group was longer than that in the rSO_2_ group, which was consistent with our result of multivariate analysis. We also found that the length of stay of MMD patients was related to hypertension, so controlling the patients' blood pressure might have a positive effect on reducing the length of stay.

Complications such as CHS syndrome and cerebral hemorrhage were low in this study, which is a decrease compared to previous studies (30, 31). The empirical method of maintaining blood pressure ~10% above the preoperative MAP before vascular anastomosis could not better provide cerebral perfusion on the affected side, even with the occurrence of cerebral perfusion syndrome and cerebral hemorrhage after the opening of the artery clamp, which was similar to the outcome of some study (11, 32). Blood pressure management should be individualized to reduce the occurrence of complications (32). Through logistic regression analysis, we found that rSO_2_ and perioperative BP control were directly correlated with postoperative neurological complications, and the BP in the rSO_2_ group was significantly lower than that in the non-rSO_2_ group, and the complications were also fewer as well. This finding suggested that intervention using rSO_2_ monitoring could assist to detect and adjust in cerebral perfusion and oxygen supply in real-time, leading to an improved prognosis for patients, as well as a shorter hospital stay, which was consistent with the results of Li et al. (33).

This study had a small sample size, and short-term follow-up and the postoperative BP management and treatment of patients in the rSO_2_ group were adjusted by the surgeon according to the value of rSO_2_. Unfortunately, we did not collect the specific management methods.

Moreover, since no monitoring of rSO_2_ was performed in the non-rSO_2_ group, no comparison of the brain oxygenation itself can be made thus putting in question whether the reduced number of complications and shorter length of stay were due to better oxygenation or purely due to the lower BP, requiring a more rigorous study design analyzing a larger sample of patients and longer follow-up to confirm or negate the value of rSO_2_ monitoring.

The current study outcome found that the intraoperative use of rSO_2_ monitoring was associated with a positive impact on the quality of recovery of patients after vascular reconstruction for ischemic MMD.

In conclusion, during the surgical procedure in patients undergoing superficial temporal anterior-middle cerebral arterial branch anastomosis for ischemic MMD, the application of MAP and rSO_2_ monitoring reduced the MAP of patients during the middle and early postoperative period and was associated with the occurrence of postoperative neurological complications compared with the patients who had only MAP detection. It is recommended to monitor rSO_2_ as a routine procedure for patients with ischemic MMD undergoing bypass surgery, which would benefit the patients.

## Data availability statement

The original contributions presented in the study are included in the article/supplementary material, further inquiries can be directed to the corresponding author.

## Ethics statement

The studies involving humans were approved by the institutional review Committee of Peking University International Hospital approved the study (KYSQ2019-058-01). The studies were conducted in accordance with the local legislation and institutional requirements. The participants provided their written informed consent to participate in this study.

## Author contributions

XC and XQ collected the clinical data and drafted the manuscript. JW analyzed and interpreted the data. RW and LY critically revised the important knowledge content. XG and JW analyzed the data statistically. All authors read and approved the manuscript.
